# A mechanistic approach to understanding range shifts in a changing world: What makes a pioneer?

**DOI:** 10.1016/j.ygcen.2015.08.022

**Published:** 2015-10-01

**Authors:** J.C. Wingfield, J.S. Krause, J.H. Perez, H.E. Chmura, Z. Németh, K.R. Word, R.M. Calisi, S.L. Meddle

**Affiliations:** a Department of Neurobiology, Physiology and Behavior, University of California, Davis, CA, USA; b MTA-DE “Lendület” Behavioral Ecology Research Group, Department of Evolutionary Zoology, University of Debrecen, Debrecen, Egyetem tér 1., 4032, Hungary; c Barnard College at Columbia University, New York, NY, USA; d The Roslin Institute and Royal (Dick) School of Veterinary Studies, The Roslin Institute Building, The University of Edinburgh, Easter Bush Campus, Midlothian EH25 9RG, Scotland, UK

**Keywords:** Climate change, Invasive species, Range expansion, Stress, Glucocorticoids, Allostasis

## Abstract

A species’ range can be thought of as a manifestation of the ecological niche in space. Within a niche, evolution has resulted in traits that maximize fitness. Across millennia, natural oscillations in temperature have caused shifts in the geographic location of appropriate habitat and with corresponding changes in species’ ranges. Contemporary climate change and human disturbance may lead to rapid range expansion or contractions with largely unknown consequences. Birds provide an excellent case study of this phenomenon with some taxa expanding range and others contracting even to the point of extinction. What leads some populations to expand while others contract? Are there physiological and behavioral attributes of “pioneers” at the forefront of a range shift/expansion?

The concept of allostasis provides a framework with which to begin to evaluate when a species will be able to successfully expand into new habitat. This tool allows the integration of normal energetic demands (e.g. wear and tear of daily and seasonal routines) with novel challenges posed by unfamiliar and human altered environments. Allostasis is particularly attractive because it allows assessment of how individual phenotypes may respond differentially to changing environments. Here, we use allostasis to evaluate what characteristics of individuals and their environment permit successful range expansion. Understanding variation in the regulatory mechanisms that influence response to a novel environment will be fundamental for understanding the phenotypes of pioneers.

## Highlights

• Many species are expanding or contracting their geographic distribution.
• Range changes are caused by climate change, human disturbance and invasive species.
• What are the characteristics of pioneers in new habitat?
• Individuals at the leading edge appear to be highly variable.
• In range expanding songbirds responses to stress are extremely variable.

## 1 Introduction

The geographical range of a species has evolved to maximize fitness in a particular ecological niche (MacArthur, 1972). Species ranges are often highly plastic, contracting or expanding to match spatial shifts of their ecological niches (Sexton et al., 2009). Changes in geographical range following climatic variations have occurred for hundreds of millions of years (e.g. Wright and Stigall, 2013), and are being documented with current changes in the Earth’s climate (Parmesan and Yohe, 2003). In addition to inducing range shifts, global climate change has lead to increases in the frequency, duration and intensity of extreme weather events (Meehl et al., 2000, Field et al., 2012, IPCC, 2012). For example, the incidence of catastrophic weather such as floods, droughts, storms, heat waves and cold spells has risen almost 10-fold in the past 50 years (Easterling et al., 2000, Beniston and Stephenson, 2004). These environmental perturbations are further compounded by human disturbance, invasive species, changes in population dynamics, and pollution. Most organisms will face major challenges in coping with one or more of these environmental challenges in the coming decades. Rapid environmental shifts can result in either deleterious or improved conditions for an individual or population. The culmination of these changes in abiotic, biotic and anthropogenic factors may lead to geographical shifts in species’ ranges at an unprecedented rate. Predicting which populations or individuals will have the capacity to shift their range as conditions change remains a challenge. Failure to adapt or shift home range may lead to population reductions or extinction. Clearly, rapid global change has placed significant and novel challenges on organisms that may not have been experienced during their evolutionary history.

The literature on dispersal biology provides a starting point to identify phenotypes that may allow individuals to successfully explore and track emerging opportunities, and shift their range when necessary (Chaine and Clobert, 2012). To complement this literature, we suggest that the concept of allostasis, or maintenance of stability through change (McEwen and Wingfield, 2003), and accompanying physiological and behavioral coping mechanisms allow us to make predictions about the factors limiting range expansion under a variety of ecological scenarios. We argue that this approach will facilitate modeling of mechanistic approaches in turn generating biologically relevant hypotheses and predictions to enable further experimental tests to identify “what makes a pioneer” at hormonal and physiological levels in vertebrates.

## 2 Species dispersals, range shifts and introductions

Dispersal biology includes natal and breeding dispersal and can occur over different spatial scales. We considered pioneering individuals at the forefront of a range expansion of breeding distribution to be a special case of dispersal and not necessarily linked directly to post-juvenile or reproductive dispersal. To that end we follow the Bowler and Benton (2005) definition of dispersal as “any movement between habitat patches, areas of suitable habitat separated in space from other such areas, irrespective of the distance between them”. Ecological invasions have been defined similarly by Vermeij (1996) as, “the geographical expansion of a species into an area not previously occupied by that species” that is consistent with the dispersal concept. Both broad definitions describe range shifts motivated by a diversity of factors from population dynamics to climate change. We have opted to follow a dispersal-based approach in that both pioneering events and invasions can be thought of as dispersal that can occur at any time in the life cycle. Here we focus on dispersal of populations into new geographic ranges resulting from climate change and shifts in native habitat.

Range expansions take a variety of forms, can include non-breeding as well as breeding ranges, and changes in habitat use may be driven by different abiotic and biotic factors. Climate change (Parmesan and Yohe, 2003), deforestation (Addis et al., 2011), urbanization (McKinney, 2008), changes in food availability (Rolshausen et al., 2009), translocation by humans (Liebl and Martin, 2012), or exclusion by introduction of novel predators and competitors (Olson et al., 2005, White et al., 2006) can all result in species leaving historic ranges and/or colonizing new habitats.

Range shifts, broadly considered, include more than just absolute changes in the geographic area occupied by a species. Temporal shifts in how species occupy space within the existing range (such as breeding on wintering grounds or overwintering on former breeding grounds) also constitute an important change in habitat use and should be considered independently (Clark, 2010, Badyaev, 2009, Stein and Badyaev, 2011, Newman et al., 2006, Atwell et al., 2012).

Range changes may occur when the quality of old habitats deteriorate, previously uninhabitable areas become more favorable, or both simultaneously. As a result, critical features of the newly occupied habitat such as climatic conditions, food resources, and important competitors may be very similar to or different from the historic range. Given the diverse nature of range expansions, we propose that finding a mechanistic framework that can be applied across different types of shifts will be helpful for making predictions about when and how colonization attempts will be successful and when and how they will fail. The concept of allostasis, which examines individual energetic balance across fluctuating environmental conditions and periods of energetic demand (allostatic load), may provide the common currency needed to make such predictions especially from a mechanistic point of view.

## 3 What makes individuals within expanding populations pioneers or followers, and what characteristics do those that endure show?

As humans it is easy to imagine, based on popular literature, that a pioneer is epitomized by the intrepid explorer, strong and bold, pushing back frontiers despite hardships and challenges. Although some pioneers of western settlements in North America were fearless explorers, in reality the vast majority were immigrants from the Old World, vulnerable to the harsh conditions of the frontier while they attempted to “tame” a vast wilderness. This point is illustrated by a stanza of the poem “The New Collosus” (1883) by Emma Lazarus that is inscribed beneath the Statue of Liberty: “*Give me your tired, your poor/your huddled masses yearning to breathe free/The wr*etc*hed refuse of your teeming shore/Send these, the homeless, tempest-tost to me/I lift my lamp beside the golden door*”.

Who, then, are the pioneers? Are they “bold and intrepid explorers,” the “wretched refuse” of the larger population, or perhaps a diverse mixture of phenotypes? Further, are the individuals that disperse from original habitats the same as, or different from, the individuals that successfully persist in the newly colonized territory? The dispersal literature describes a three step process: dispersal from the old habitat (emigration), transition, and settlement in the new habitat (immigration) (Clobert et al., 2009, Bowler and Benton, 2005). Each step of dispersal provides a selective screen; some individuals will emigrate, few survive transition, and fewer still establish in the new habitat. Investigations showing phenotypic differences of populations in recently colonized areas versus those from sites that have been established for long periods (e.g. Duckworth and Badyaev, 2007, Hanski et al., 2004, Lindstrom et al., 2013). While the bulk of our discussion concerns the mechanistic traits that enable individuals in range expanding populations to establish and persist in new habitat, next we briefly address recent findings on the dispersal phase of range change. We highlight these examples to emphasize that “successful pioneers” must arise from a subset of the individuals that leave the historic range.

Behavioral traits associated with dispersal include aggression (e.g. Duckworth and Badyaev, 2007), exploration (e.g. Dingemanse et al., 2003), and sociability with asocial individuals dispersing further (e.g. Cote et al., 2010). Furthermore, the behavioral traits of a disperser may differ across contexts: dispersers from high density populations tend to be asocial, while dispersers from low density sites are generally more social (Cote and Clobert, 2007). The physiological or morphological traits and mechanisms underlying behavioral differences in dispersing individuals are not well known. The first cane toads, *Rhinella marina*, to arrive on new sites of an invasion front in Australia had longer legs than individuals in established populations (Phillips et al., 2006). Additionally, condition-dependent dispersal appears also to be important though directionality differs across systems (reviewed in Clobert et al., 2009). In some cases, individuals in poorer condition have a higher propensity for dispersal (Bowler and Benton, 2005, Roff and Fairbairn, 2001, Sinervo et al., 2006). Studies in naked mole rats (*Heterocephalus glaber*), side-blotched lizards (*Uta stansburiana*) and Belding’s ground squirrels (*Urocitellus beldingi*) have shown that dispersing individuals often have a higher body mass than non-dispersers (Bowler and Benton, 2005, Sinervo et al., 2006, Holekamp, 1986, O'Riain et al., 1996). However, mechanisms remain to be determined. A few investigations have described distinctive patterns in testosterone, glucocorticoids, and serotonin secretion in dispersers (reviewed in Clobert et al., 2009). While the population of individuals that settle in new habitat must be drawn from those individuals that first disperse, it is important to note that mechanistic traits facilitating dispersal may, or may not, be the same traits that facilitate establishment in the new territory following range expansion.

## 4 The concept of allostasis and the emergency life history stage

How then will individual traits that promote dispersal and establishment, which also requires maintenance of homeostasis, influence success within a novel environment? Within in any life history stage, homeostasis is tightly regulated to maintain energetic balance. However, it would be inappropriate to assume that homeostatic levels should be maintained at a constant set point throughout the annual cycle because an animal experiences different energetic demands at each life history stage. Allostasis is the process by which homeostatic set points are adjusted to meet the predictable and unpredictable demands of each life history stage within the annual cycle through physiological and/or behavioral processes. Furthermore, if energetic demands increase above available energy then the current life history stage is disrupted and the emergency life history stage is triggered to allow the individual to cope until the perturbation passes, or the animal moves to a more favorable environment (e.g. Wingfield, 2003, Wingfield et al., 1998).

**Fig. 1:**
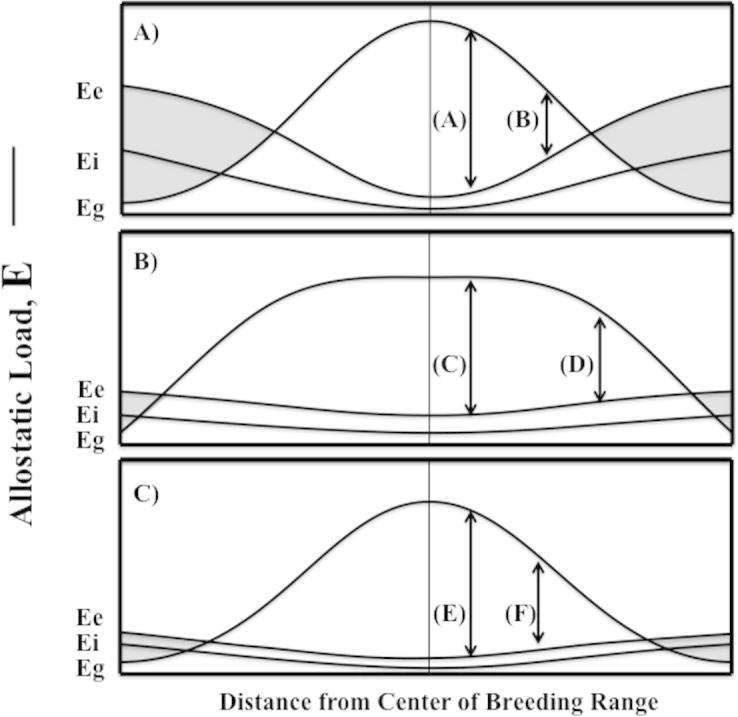
The allostasis model can be used to predict a population range. Animals have evolved to increase overall fitness within a particular niche. As an animal moves from the center (indicated by vertical line) of the range to the periphery, the habitat may decline in quality due to a decrease in energy that can be gained from the environment (Eg). In addition, changes in temperature and or habitat lead to adjustments in resting metabolism (Ee) and routine metabolism (Ei). The arrows immediately adjacent to the vertical line indicate an excess of Eg relative to Ee + Ei (perturbation resistance potential, PRP). As an animal moves away toward the periphery of the range, represented by the right hand arrow, the excess of Eg is greatly reduced. The intersection of Ee and Eg is the critical point were an animal would no longer be able to maintain energy balance and would enter into allostatic overload type I (AOL, shaded area). On the top panel A) arrow (A) is greater than (B) showing reduced PRP toward the periphery of geographic range. In the center panel B), Eg remains high over much of the range and drops off quickly near the periphery. Arrows (C) and (D) show less reduction in PRP. In the bottom panel C) Eg is as for the top panel, Ee and Ei decrease much less toward the periphery but arrows (E) and (F) still show reduced PRP. Negative energy balance is not sustainable and thus colonization is unlikely or impossible. See Table 1 for definitions of terms.

The concept of allostasis provides a heuristic framework for directly assessing daily individual energetic demands by factoring in everyday routines, gender, age, social status, injury, parasite load, etc.; in relation to food available in the environment and individual energy stores that can be drawn upon to fuel that load (Table 1, Fig. 1, McEwen and Wingfield, 2003, Wingfield, 2004, Goymann and Wingfield, 2004, Korte et al., 2005). Using this concept, we can examine two critical components of a range expansion/shift scenario: the energetic impact of the transition from old to new geographic range, and the energetic advantages or disadvantages associated with phenotypic variation that might be present in the pioneering individuals.

Allostatic load defines the total amount of energy used by an individual to meet daily energetic needs, termed E (Table 1). E can be subdivided into components that may fluctuate with the annual cycle. These include the existence energy (Ee, Table 1) required to maintain life at different times of year (basal metabolic rate; BMR) and is primarily influenced by temperature as well as general wear and tear. An example of such wear and tear effects would be increased Ee as a result of degraded or missing feathers, which impact thermoregulatory capacity. Routine energy expenditure (Ei) is the extra energy required to go about daily routines of foraging, breeding, migration, etc. throughout the year (Table 1). Next is the energy required to cope with non-routine perturbations of the environment (Eo, Table 1). Finally, an individual’s allostatic load can be compared with the total amount of energy that can be gained from the environment while also taking into account metabolic energy stored in the form of protein, lipids and carbohydrates that can be utilized via catabolic processes to temporarily buffer reductions in energy gained (Eg, Table 1). The difference in Eg and E has been termed the perturbation resistance potential where sufficient energy can be gained from the environment to support increasing metabolic needs before negative energy balance occurs.

Allostatic overload occurs when the energy expended by an individual exceeds the energy gained from the environment (Eg < Ee + Ei + Eo, Table 1) resulting in negative energy balance. A key issue is what happens when an individual experiences allostatic overload? Typically, the emergency life history stage (ELHS) is activated resulting in the interruption of the life history stage appropriate for that time of year, and mobilizing energetic resources to allow an individual to cope with the perturbation and promote immediate survival. For example, in some cases as the condition of allostatic overload threatens this may be sufficient to result in nest abandonment and thus reproductive failure. Note that the EHLS can be activated at any time of the year and in any life history stage (Wingfield, 2003, Wingfield, 2013a, Wingfield, 2013b). However, one individual may activate the ELHS, interrupt the current life history stage and even depart the area, while another facing the equivalent energetic challenge but in a territory/range that is higher quality is able to avoid allostatic overload and remains in the current life history stage.

## 5 The Allostasis framework and predicting the likelihood of colonization leading to range expansion

In order to better understand the likelihood of whether or not a pioneer will persist and ultimately colonize an area, we explore the overall energetic balance of a pioneer in a novel geographic range (Fig. 1). The most critical permissive component of the allostasis framework that determines if an individual can colonize a novel region is the total energy that can be gained from the environment (Machmer and Ydenberg, 1990, Costa, 2007, Costa, 2012) integrated with the energy stored by the individual (Eg). Climate is one variable that may affect Eg as it alters food production, water supply, and habitat; important factors that structure the fundamental niche in which an organism can survive. While habitat quality can be patchy throughout a species range, in general it is thought to decline towards range limits (Sexton et al., 2009). Studies by Gross and Price (2000) on Hume’s leaf warbler, (*Phylloscopus humei*), showed a positive relationship between arthropod abundance and distribution at the northern limit of the populations range while the southern limit was suspected to be limited by increased competition. A similar study, conducted on 85 species of finch living in three different countries found strong positive relationships between food abundance and bird densities. The authors also found that population densities were reduced by 75% on the mainland compared to the Galapagos which they attributed to increased predation and competition for food resources (Schluter and Repasky, 1991). In Fig. 1, we indicate that Eg declines from the center towards the periphery of the range as either total food availability changes or the ability of animal obtain and/or assimilate it declines. In addition to potential reductions in Eg, Ei increases toward the periphery of the range due to one or combinations of the following: increased parasite load, predation pressure, heterospecific competition, when morphological traits for locomotion or foraging becomes a disadvantage and others. Ee will also increase if ambient temperature is outside the thermal neutral zone (Kendeigh, 1969).

At the edge of a population’s range, the habitat becomes less suitable to the point where negative energy balance is reached due to the combination of a decline in Eg, and elevations in Ee and Ei. In Fig. 1, entry into negative energy balance occurs at the intersection of Eg and Ei + Ee and is the threshold for allostatic overload. Therefore the likelihood that a pioneer will colonize a novel area is dependent upon the perturbation resistance potential (i.e. Eg − E) (Wingfield et al., 2011a, Wingfield et al., 2011b). This perturbation resistance potential varies with changes in the model components illustrated by the vertical arrows in Fig. 1. For individuals of a range expanding population, the ability to maximize perturbation resistance potential will be an important determinant of who colonizes new geographic range and who may perish. Examples of field studies which support these predictions include facultative movements from high to low elevations by mountain white-crowned sparrows, *Zonotrichia leucophrys oriantha* in relation to snow storms in early spring in order to reduce Ee + Ei associated with decreased temperatures and increased foraging effort while also taking advantage of greater Eg due to reduced snow cover (Breuner and Hahn, 2003).

## 6 Allostasis and survival of colonizing pioneers in novel geographic ranges

**Fig. 2:**
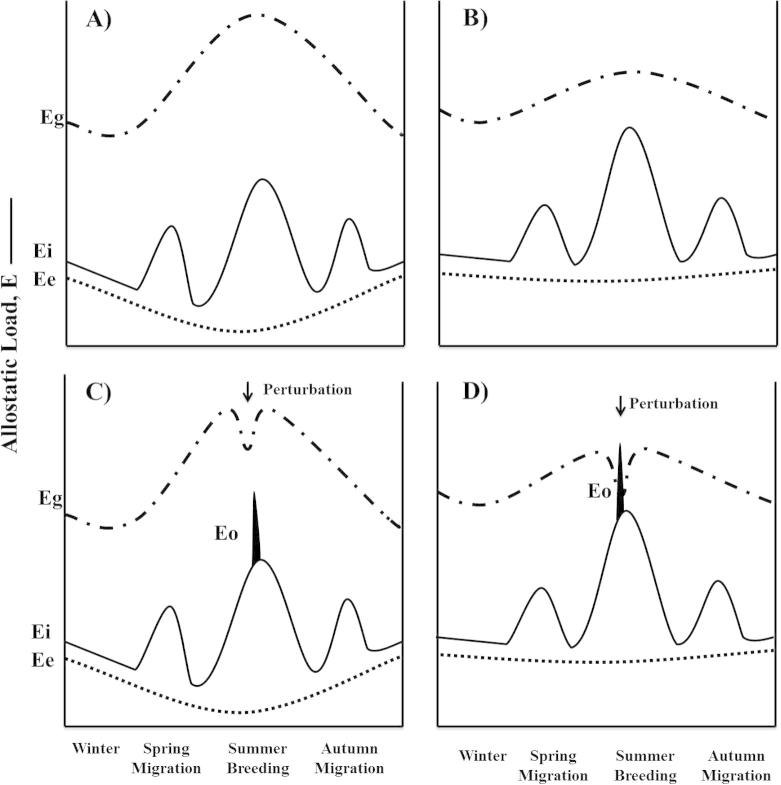
The four captions depict natural fluctuations in energy gained from the environment and changes in metabolic energy expenditure as, for example, a migratory bird proceeds through the annual cycle. Caption A depicts a bird that is in an optimal habitat that allows for fluctuations in Ee and Ei, associated with different life history stages such as migration and breeding because Eg remains high. In this scenario, allostatic overload is unlikely. Caption B depicts a bird that is in a suboptimal habitat where there fluctuations in Ee and Ei reach greater levels, and Eg is lower. In this scenario the bird is more vulnerable to any perturbations of the environment that may occur and further reduce Eg, and/or increase Ee + Ei. The ability of the individual in B to cope with environmental perturbations will be drastically different from the individual in A (i.e. resistance potential is reduced per Wingfield et al., 2011a, Wingfield et al., 2011b). In caption C a perturbation of the environment (such as a storm represented by the black peak) requires the bird to expend additional energy to cope (Eo), but nonetheless is able to stay in positive energy balance even though Eg is also decreased. The bird in caption D has to cope with the same perturbation (identical dimensions of the black peak), but because Ee + Ei is also higher it is unable to maintain energy balance resulting in allostatic overload. The ability of a pioneer to avoid instances of allostatic overload (i.e. it has high resistance potential, Wingfield et al., 2011a, Wingfield et al., 2011b) will determine the success rate for colonizing a new geographical area. The resistance potential is, therefore, a function of Eg in relation to Ee + Ei + Eo on a day-to-day basis. For definitions of terms see Table 1.

Eg can vary dramatically over the year and energetic requirements for Ee + Ei fluctuate as birds migrate, molt, breed, etc. Eg, Ei and Ee that a pioneer experiences may differ greatly due to individual characteristics as well as habitat characteristics that may vary by individual territory such as competition with other species, temperature, predation, territory quality, etc. (Goymann and Wingfield, 2004, Korte et al., 2005, Wingfield, 2004). Fig. 2, represents potential scenarios that two pioneering individuals might encounter. These are characterized by one individual showing a decrease in overall Ee + Ei during the warmer months of spring and summer with an increased Eg that more than compensates for fluctuating costs of migration, breeding, etc. (Fig. 2A) and is compared to an individual that experiences larger fluctuations in Ee and Ei combined with a lower Eg (Fig. 2B). The additional allostatic load incurred by the individual (Fig. 2B) may be due to a suboptimal habitat or individual characteristics that make daily routines more energetically expensive. In the absence of an environmental perturbation, there is sufficient energy in the environment (Eg) to ensure survival and reproductive success in both scenarios. However, in many years, most individuals are likely to experience perturbations of their environment such as weather events, food shortages, predation attempts, competition, social disputes, etc. (Wingfield et al., 2011a, Wingfield et al., 2011b) which can lead to reductions in Eg as depicted in Fig. 2C and D. A classic example can be taken from the Arctic when snow storms blanket the landscape and reduce access to food for songbirds (Astheimer et al., 1995). Both individuals (in Fig. 2) must increase their energy expenditure to cope with Eo, which is above and beyond Ei + Ee. Increased foraging effort to uncover food after, for example, a snow fall contributes to Eo, lower temperature elevates Ee and snow cover decreases Eg. Even though Ee + Ei and Eo are elevated in response to the storm there is adequate Eg to prevent allostatic overload in the first individual (Fig. 2C). In the other scenario where Eg is lower to begin with and Ee + Ei elevated in responses to the storm, then the same perturbation would result in allostatic overload because of reduced resistance potential. Entry into allostatic overload would force these individuals to abandon breeding or other life history stages to favor coping strategies of physiology and behavior that promote survival (Emergency Life History Stage, Wingfield, 2003, McEwen and Wingfield, 2003, Korte et al., 2005, Fig. 2D). Thus we would predict that a pioneering individual similar to 2A and 2C would be likely to colonize and persist in the new habitat while the second individual would be at higher risk of failing.

## 7 Allostasis and reproductive success of colonizing individuals

**Fig. 3:**
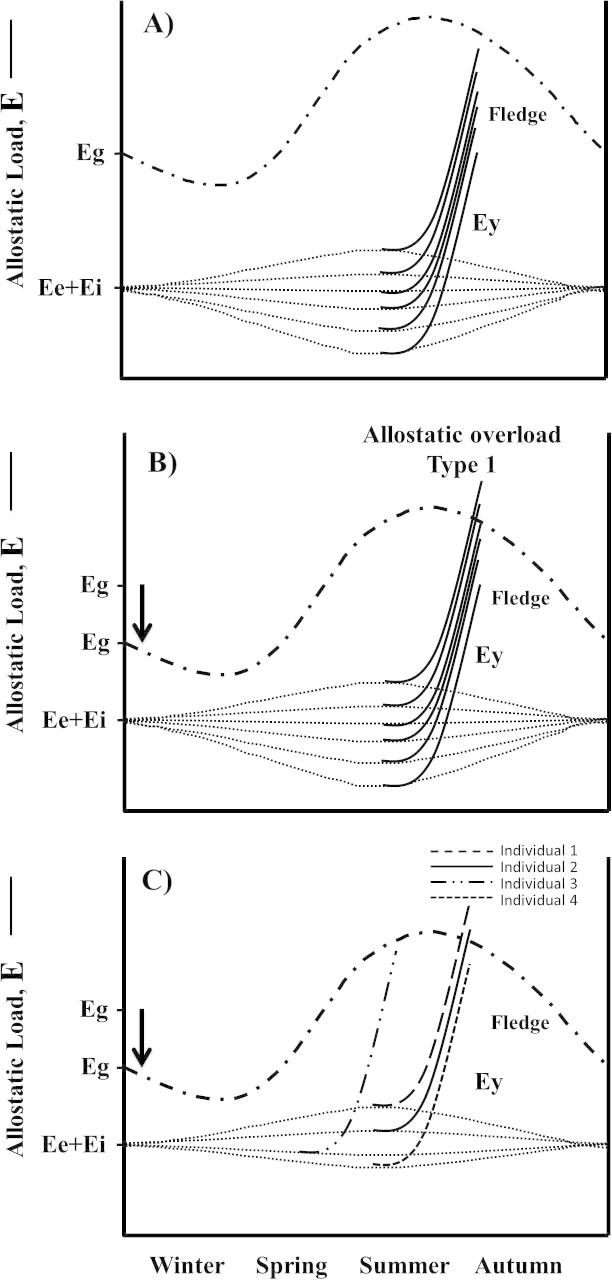
Caption A shows individual differences in Ee + Ei and implications for Ey (allostatic load accrued from raising young to independence – fledging). In this scenario, Eg is sufficient for all individuals to raise young, although those with the greatest Ee + Ei + Ey will be more vulnerable to an unpredictable event that may elevate allostatic load even further (i.e. lower perturbation resistance potential). Caption B, Eg is decreased as might occur with climate change or if invading new range. Now, those with the greatest Ee + Ei + Ey will develop allostatic overload and reproductive failure follows. However, Caption C, if there is individual variation in the timing of egg laying, individuals that may not otherwise succeed may do so if their timing better matches availability of resources in the new habitat.

The concept of allostatic load (E) may be further applied to the question of whether or not a pioneering individual will successfully reproduce in a novel habitat. Because reproductive costs are often facultative and incur a large and temporally concentrated allostatic load, it may be useful to consider an additional energetic term in the model, Ey that incorporates the energetic demands of both parent and offspring Ei + Ee (Fig. 3). Allostatic load increases as foraging rates rise to meet the metabolic demands of growing nestlings. Meeting such demands is a requirement of reproductive success. If we introduce a line for Ey and apply it to each individual, in Fig. 3A, all individuals are able to fledge young successfully despite increased allostatic load of breeding due to a seasonally high Eg. In another habitat where Eg is lower during the breeding season, some individuals with higher Ey exceed Eg and reach allostatic overload which results in nest abandonment (Fig. 3B) However, in addition to individual variation in Ee and Ei, there may also be individual variation in the timing of the onset of reproduction. If this is the case, a pioneer may be able to take advantage of greater Eg by adjusting the match between timing of onset of breeding, particularly the nestling stage and peak food availability. This scenario is illustrated in Fig. 3C, in which an individual that initiates reproduction earlier does not encounter overload while an individual that maintains initiation at the average time does, even though all other individual parameters may be the same. Thus both individual variation in allostatic load and timing of reproductive events will determine reproductive success if Eg is lower but temporally variable in a new range.

## 8 Physiological mechanisms used by pioneers in colonizing novel ranges

### 8.1 Hormonal mechanisms

There are numerous mechanisms, physiological, neural, immunological and behavioral, that enable organisms to cope with both predictable and unpredictable environmental variation. Major candidates for the physiological mediation of range expansion are growth factors, cytokines, thyroid hormones and glucocorticoids among others. These systems have well characterized roles in regulating processes in relation to energetic demands and associated behavior (Wingfield and Romero, 2001, Wingfield, 2003). Collectively these and other regulatory hormones are the mediators of allostasis, comprising the reactive scope of the organism in response to environmental change (Romero et al., 2009). The relationship between the mediators and changes in allostatic load remains largely unknown.

**Fig. 4:**
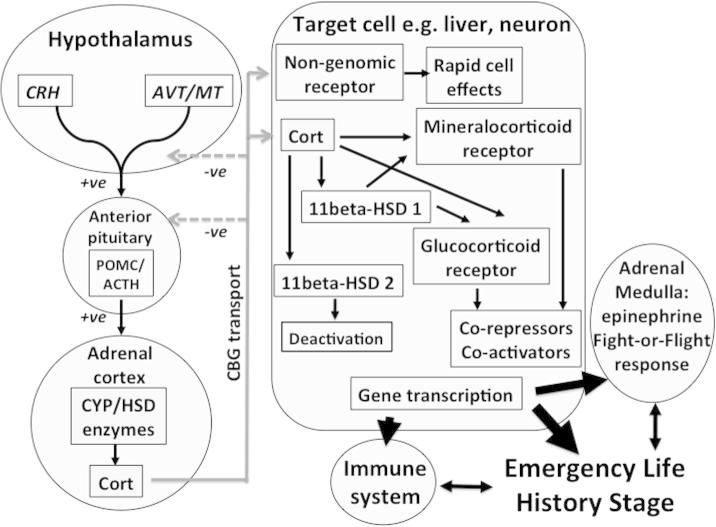
The simplified three part regulatory system of secretion control, transport and hormone responses: the adrenocortical response to acute environmental perturbations that trigger an emergency life history stage (ELHS). The left hand part of the figure represents the hormone secretion cascade, in this case the hypothalamo–pituitary–adrenal cortex (HPA) axis. Perturbations of the environmental are perceived by sensory modalities and that information is transduced into neuropeptide secretions such as corticotropin-releasing hormone (CRH), arginine vasotocin (AVT) and mesotocin (MT) that regulate expression of a precursor or pro-peptide hormone, pro-opiomelanocortin (POMC) in the anterior pituitary corticotrophs. POMC can be then cleaved to give several peptides including adrenocorticotropin (ACTH). Release of ACTH from the pituitary gland into the blood is also regulated by CRH and AVT. ACTH acts on adrenocortical cells to activate CYP enzymes including hydroxysteroid dehydrogenases that synthesis glucocorticoids such as corticosterone (Cort). Release of Cort into the blood is a major end point of the cascade of events that are part of the adrenocortical response to stress. Once in the blood, Cort circulates bound to a carrier protein corticosteroid-binding globulin (CBG) which is part of the transport part of the system ( gray lines). Cort provides negative feedback signals for ACTH release from the pituitary as well as CRH release from the hypothalamus. More than 90% of Cort circulating in avian blood is bound by CBG. On reaching target cells such as liver or neurons in the brain involved in the emergency life history (coping) stage, it is thought that only Cort unbound to CBG can enter cells. Once inside the cell there are two types of genomic receptor that can bind Cort and become gene transcription factors. The mineralocorticoid type receptor (MR) binds with high affinity and so can be saturated at low levels of Cort. The glucocorticoid type receptor (GR) has a lower affinity for Cort and is saturated only at higher concentrations of Cort. Thus GR has been proposed as the “stress” receptor. Note also that there is strong evidence for a membrane receptor (non-genomic) that mediates rapid behavioral effects within minutes. The genomic receptors have effects through gene transcription, different genes affected by each receptor type) and thus require up to several hours for biological effects to be manifest. As seen in Fig. 1, Fig. 2, there are also steroidogenic enzymes expressed in target cells that can modulate how much Cort encounters at least genomic receptors. 11beta-hydroxysteroid-dehydrogenase (11beta-HSD) has two major forms – 1 and 2. 11beta-HSD 2 converts corticosterone to deoxycorticosterone which cannot bind to any known Cort receptor and is a deactivation shunt. 11beta-HSD 1 tends to have the opposite effect enhancing Cort and thus likelihood of binding to MR or GR. As in Fig. 1, Fig. 2, co-repressors and co-activators also are points of regulation for gene transcription and responses that control the emergency life history stage and affect the immune system. The adrenal medulla is a key component of the fight-or-flight response (right hand part of the figure) secreting epinephrine and nor-epinephrine. This neuroendocrine system is also involved in the emergency life history stage. This three part system, hormone cascade, transport in blood and response networks in the target cells is also well conserved across vertebrates, but the diversity of ways by which specific components can be regulated to modulate responsiveness to stress is very great. From Wingfield, 2013a, Wingfield, 2013b, courtesy of the Ecological Society of America.

There is growing evidence that increasing allostatic load is accompanied by elevations of glucocorticoid secretion through activation of the hypothalamo–pituitary–adrenal (HPA) axis (Fig. 4), particularly when allostatic overload occurs (e.g. McEwen and Wingfield, 2003). These responses are consistent with the well established adrenocortical response to stress that results in increases in plasma glucocorticoids to stressors in general (e.g. Wingfield, 2001, McEwen and Wingfield, 2003, Romero et al., 2009). In recent years, growing evidence indicates that the adrenocortical responses to acute stress are modulated at both the population level in relation to season, altitude and latitude as well as at the individual level in relation to age, sex, social status, and individual experience (Wingfield et al., 1995, Wingfield, 2001, Wingfield, 2005, Wingfield, 2012, Wingfield, 2013a, Wingfield, 2013b, Wingfield and Romero, 2001, Wingfield and Sapolsky, 2003). Furthermore, there is a strong tendency for individuals to increase adrenocortical responses to acute restraint stress at the limits of their breeding ranges (e.g.; Dunlap and Wingfield, 1995, Wingfield, 2005, Addis et al., 2011, Liebl and Martin, 2012, Walker et al., 2015, Krause et al., 2015). Activation of the HPA axis and the resulting rise of corticosteroids enable organisms to cope with stressors, altering behavior and physiology to promote survival. Given that there is individual variation in ability to modulate the adrenocortical responses to stress, it is important to consider that rapid variation of circulating levels of corticosterone reveals considerable phenotypic flexibility in the face of coping with variable environments.

It should be noted that other components of the HPA axis such as binding proteins that transport corticosteroids in blood, and receptors and metabolizing enzymes in the target tissues, also provide potential for individual variation in mechanisms of modulation (e.g. Wingfield, 2012, Wingfield, 2013a, Wingfield, 2013b; Fig. 4). Could individual variation in the modulation of the adrenocortical responses to stress and allostatic load in general be characteristic of populations expanding/changing their ranges? Do pioneers modulate the HPA axis differently from non-pioneering individuals? Is it also possible that increased frequency and intensity of perturbations of the environment, associated with climate change, result in a mismatch of the adrenocortical response of some individuals to acute stress and the new environmental conditions (Angelier and Wingfield, 2013). These questions allow development of hypotheses that can be tested in the field and laboratory across a broad spectrum of vertebrate species. Several potential hypotheses (H) and associated predictions (P) are:

H1: Modulation of adrenocortical activity (baseline, maximum and total corticosteroid released) in relation to resistance potential encountered in a novel habitat is key to success for individuals at the leading edge of a range.

P1: Adrenocortical responses to environmental perturbations are up-regulated in individuals with low resistance potential.
P2: Adrenocortical responses to environmental perturbations are down-regulated in individuals with high resistance potential.

H2: Adrenocortical activity characteristic of an individual should match the environmental conditions/challenges it encounters in order for it to be successful at the leading edge of a range.

P1: If an organism mounts a suboptimal (i.e. over- or under-activate) adrenocortical response to a perturbation, it will exhibit inappropriate behaviors for the challenge encountered and fail to return to homeostasis leading to decreased fitness.
P2: If an organism mounts an optimal adrenocortical response that matches the level of the disturbance it encounters it will exhibit behaviors and physiological changes that effectively return the organism to homeostasis, enhancing fitness.

### 8.2 Examples from the field

With the hypotheses and predictions above now in mind, we review examples from recent field studies, mostly in birds. These studies also raise issues about what data needs to be collected, when, and in what populations? Work has already begun both directly and indirectly to explore these and related questions regarding the mechanistic underpinnings of range change.

In a population of house sparrows, *Passer domesticus*, in Kenya expanding inland along corridors of human development (roads), the birds at the leading edges of the range expansion showed greater responsiveness of the adrenocortical responses to acute stress and enhanced exploratory behavior. However, these effects were only manifested in breeding birds (Liebl and Martin, 2012). Furthermore, hippocampal mRNA expression for mineralocorticoid receptors (MR) were lowest in relation to glucocorticoid receptors (GR) in house sparrows at the leading edge of the range expansion (Liebl and Martin, 2013). It would be interesting to determine Eg available and allostatic load in these leading edge individuals especially because they are in human disturbed areas.

**Fig. 5:**
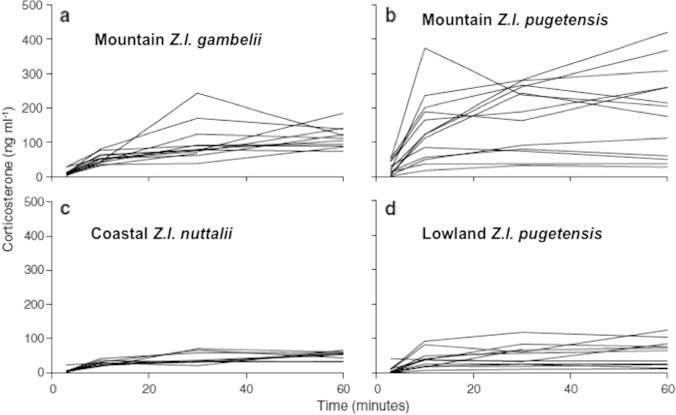
Individual integrated plasma corticosterone responses for different population of white-crowned sparrows, *Zonotrichia leucophrys*. Variance is different between populations (Levene’s test *p* = 0.003). (a) *Z. l. gambelii*; (b) Highland *Z.l. pugetensis*; (c) *Z.l. nuttalli*; and (d) Lowland *Z.l. pugetensis.* From Addis et al. (2011), courtesy of Springer, Berlin. Caption b shows the variation in adrenocortical responses to stress in a population that is extending its range.

In recent decades the Puget Sound white-crowned sparrow, *Z.l. pugetensis*, has established breeding populations in urban habitats and has also expanded its range to human-disturbed habitat in clear cuts of mountain forests and in ski areas in alpine zones of the Cascade Mountains of Washington State. These birds at the leading edge of an expansion of breeding range show extreme individual variation in profiles of adrenocortical responses to acute stress compared with individuals in ancestral habitat or with individuals of other sub-species naturally breeding in alpine meadows (Addis et al., 2011; Fig. 5). This is consistent with Hypothesis 1, Prediction 1, but follow up information is needed to determine if the individual variation in the adrenocortical responses to acute restraint stress decreases as selection favors those phenotypes that persist in the population.

In contrast, a migratory population of dark-eyed juncos, *Junco hyemalis thurberi*, breeding in the mountains of Southern California and wintering along nearby coastal regions, recently established a non-migratory breeding population in urban wintering habitat (Newman et al., 2006). Juncos that had recently colonized the urban wintering habitat to breed showed reduced adrenocortical responses to acute stress (nesting females only) and bolder exploratory behavior compared to breeding juncos in the original mountain habitat (Atwell et al., 2012). Furthermore, these differences persisted in the two populations brought into captivity in a common garden experiment (Atwell et al., 2012). The reduced adrenocortical responses to acute restraint stress in nesting female juncos is unlike house sparrows and male Puget Sound White-crowned sparrows above. The junco example could be consistent with hypothesis 1 prediction 2 as well as hypothesis 2. However, Bonier et al. (2006) showed that in *Z.l. pugetensis* that had colonized urban habitats in Washington State and California, nesting females showed a non-significant trend for reduced adrenocortical responses to acute stress. Note that males revealed a significant increase in the responses of the HPA axis, more consistent with hypothesis 2. Clearly sex differences must be taken into account and there may be examples for all of the predictions made above, as well as others as yet unknown.

Given these results, it appears possible that both population and individual variation in some or many traits may be characteristic of populations that are pioneers in colonizing new range. Selection would then act on the subset of phenotypes that are able to both persist in the new environment and successfully raise young. The allostasis framework may have heuristic value because individual variation in costs of daily and seasonal routines, Ee + Ei, may determine how well they fare when encountering new habitats or competing with novel species. In other words, those individuals with greatest resistance potential and are best able to avoid allostatic overload type1 will cope better with new habitat and the novel environmental perturbations that could also result. More comparative studies, particularly in the field, are urgently needed.

### 8.3 Other physiological mechanisms

Though our main focus has been on glucocorticoids as mediators of range change, here we will highlight, briefly, a few additional mechanisms that may prove fruitful for future investigation. Much more work is also needed and we do not attempt to review the potential alternate mediators here. Immune function has been shown to vary both temporally and between individuals and Lee and Klasing (2004) suggest that variation in immunocompetence may be important for range change, as novel environments will present differences in immune requirements. These differences will determine how resources may be allocated to immune responses versus life history stages (allostatic load) such as reproduction and survival following perturbations of the environment (Lee and Klasing, 2004). These variable demands will interact with the unique phenotype of the individual to determine fitness in the new environment. The application of the allostasis framework in these scenarios is unexplored except for preliminary indications that the costs (allostatic load) of mounting an immune response are critical (e.g. Ashley and Wingfield, 2011).

As regulators of numerous metabolic and seasonal processes the thyroid hormones triiodothyronine (T3) and thyroxine (T4) may play critical roles in an organism’s ability to adjust to a novel habitat. They may be key for coping with allostatic load including possible variation in TH receptor expression patterns that may have variable effects depending on the environmental challenges encountered. Thyroid hormones play an important role in regulating thermogenesis in response to variable temperature, however the directionality of response varies depending on conditions (Deligiannis et al., 1993). Increased T3 expression has been shown to stimulate food intake (Leblond and Gross, 1943, Tomasi and Horwitz, 1987, Hesslink et al., 1992, Ishii et al., 2008). Thus variation in the hypothalamo–pituitary–thyroid axis (HPT) activity may facilitate mobilization of energy stores for temporary reduction of allostatic load in novel environments. Furthermore, thyroid hormone signaling has been implicated in control of the timing of vernal life history stages in several species of photoperiodic birds (e.g. Pathak and Chandola, 1982, Yasuo et al., 2005, Nakao et al., 2008, Pérez & Wingfield unpublished data). Variation in the HPT axis thus might allow for enhanced matching of novel environments by adjusting timing of major life history events.

Finally, in the shorter term scale of a range expansion front, recent work has suggested that epigenetic mechanisms may serve to generate additional phenotypic flexibility and adaptation. Gene methylation patterns were variable in house sparrows undergoing range expansion in Kenya and may increase phenotypic variation and/or flexibility as new environments are encountered (Liebl et al., 2013). This in turn would be an important source of individual variation leading to adaptation over the abbreviated time scales in which invasions occur.

## 9 Conclusions and future directions

The success of pioneering individuals and populations is dependent not only on the phenotype of the pioneer, but the interaction between phenotype and the novel environment. The framework of allostasis provides a method for linking organism–environment interactions to physiological processes. We suggest that integrating an understanding of the physiological mechanisms associated with range expansion will enhance ecological and evolutionary studies of range expansion allowing more robust predictions. For instance, Badyaev (2009) demonstrated that an intimate knowledge of incubation physiology helps to explain the production of novel phenotypes and subsequent phenotypic accommodation during a range expansion of house finches, *Haemorhous mexicanus*, in Montana. The potential benefits from integrating physiology into such studies are only beginning to be realized.

Understanding the kinds of phenotypic variants that can be produced in response to novel environmental cues requires a deep understanding of underlying organismal physiology (Németh et al., 2013, Wingfield, 2013a). Future work should seek to address several key questions. How do mediators of allostasis vary between individuals/populations whose ranges are expanding, contracting or remaining unchanged? Do pioneers have traits that give them an advantage? How do these mediators change throughout the range and are they specific to pioneers? Are there consistent patterns across species and contexts that can be used predictively? Additionally, how do invasive species that are successful after artificial introduction compare with individuals of populations that are shifting range naturally after environment changes? Taking a mechanistic approach, such as allostasis, may provide specific and testable predictions to answer these questions.

## Figures and Tables

**Table 1:** Environmental factors and allostasis, definitions and examples.

Term	Definition	Direction of change that is challenging	Environmental factors that contribute to the challenge	Individual phenotype that could resolve or counteract the challenge
Ee	Existence energy (Resting Metabolic Rate)	Increase	Temperature above or below thermoneutral zone	Changes in insulation, size, use torpor, thermoregulation, and metabolic efficiency. Innovation in finding shelter
Ei	Daily routines above existence energy (Routine Metabolic Rate)	Increase	Increased competition, predation, or decreased food distribution/availability/or change in food type (related to Eg) will increase Ei and Ey	Decreased reproductive output, shorter lifespan, eliminating overlap between life-history stages, innovation in anti-predator behavior. Behavioral phenotypes that enhance foraging efficiency (also affect Eg)
Ey	Energy to raise offspring to independence	Increase
Eo	Energetic cost due to perturbations in the Environment or non-routine events	Increase	Added cost over Ei to overcome a perturbation. (e.g., storms, predation, social disputes, natural or human-induced disasters, parasites or disease	Larger individuals, larger fat stores, efficient foragers, bold, etc
E	Allostatic load; Overall energy expenditure (E = Ei + Ee + Eo + Ey)	Increase	Determined by fluctuations in Ei, Ee, and Eo. Net effect of increase determined by Eg	
Eg	Energy available (Gained) from environment	Decrease	Decreased food availability, which may be due to reduced amounts or restricted access due to weather (e.g. snow cover), competition, or foraging efficiency/time	Behavioral phenotypes that enhance foraging efficiency, competitive advantage for resources, shifts in timing of life-history stages that better match fluctuating Eg

Positive/Neutral Energy Balance: Eg ⩾ Ee + Ei + Eo; Negative Energy Balance/Allostatic Overload: Eg < Ee + Ei + Eo.

From McEwen and Wingfield, 2003, McEwen and Wingfield, 2010, Wingfield, 2013a, Wingfield, 2013b.
